# Contrast Sensitivity Function in Parkinson’s Disease: Effects of Illumination and Disease Stage

**DOI:** 10.1007/s44402-026-00091-7

**Published:** 2026-04-27

**Authors:** Alba Herrero-Gracia, Rosa Hernández-Andrés, Carlos Perla-Muedra, Kenneth J. Ciuffreda, Mª Amparo Díez-Ajenjo

**Affiliations:** 1 Department of Optics and Optometry and Vision Sciences, Faculty of Physics, University of Valencia, Valencia, Spain; 2 Neurology Department, Arnau Vilanova-Llíria Hospital, Valencia, Spain; 3 Department of Biological and Vision Sciences, College of Optometry, State University of New York, New York, New York USA; 4 Fundación de Oftalmología Médica, Valencia, Spain

**Keywords:** Contrast sensitivity function, Hoehn and Yahr stage, Illumination conditions, Parkinson’s disease, Visual function

## Abstract

**Purpose:**

Contrast sensitivity function (CSF) is a valuable tool for assessing visual function, which can be affected in neurodegenerative disorders such as Parkinson’s Disease (PD). Visual perception in everyday life depends on the ability to detect objects across a wide range of spatial frequencies under different illumination conditions. However, the effect of disease progression and varying illumination on CSF in individuals with PD remains underexplored. The aim of this study was to investigate CSF in PD participants across six illumination conditions, examining disease stage influence and comparing results with age-matched healthy controls.

**Methods:**

CSF was measured with the Functional Acuity Contrast Test. This prospective, comparative study evaluated CSF under photopic, mesopic and glare conditions, across disease stages, classified according to the Hoehn and Yahr Scale. The index of contrast sensitivity (ICS) assessed overall CSF.

**Results:**

Seventy-one PD participants and 97 healthy controls were included. PD participants demonstrated significantly poorer CSF than controls across photopic, mesopic and glare conditions (mixed-model analysis, *p* < 0.001). The most pronounced impairment was observed under mesopic conditions with mild glare (ICS: 33 vs. 2.5, *p* = 0.02). Unadjusted analyses suggested a decline in CSF with advancing disease stage; however, this effect was attenuated after controlling for age. No significant relationship was found between disease progression and ICS after age adjustment under any illumination condition.

**Conclusions:**

This is the first study to assess CSF comprehensively with ICS data under six illumination conditions in PD, considering disease stage and comparing with controls. PD participants experience significant visual deficits, particularly under mesopic and glare conditions. While contrast impairments are robust in PD, stage-related differences appear partially influenced by age. These findings highlight the clinical importance of evaluating CSF under ecologically relevant lighting environments to better characterise functional visual impairment in PD and its impact on activities of daily living.

Key Points

- Participants with Parkinson’s Disease exhibit a significantly poorer contrast sensitivity function than controls across photopic, mesopic and glare conditions.
- The impaired contrast sensitivity function is most pronounced under mesopic conditions in participants with Parkinson’s Disease, where it was significantly worse than in controls.
- Differences in contrast sensitivity function across disease stages were observed in unadjusted analyses, but stage-related effects were attenuated after controlling for age.

## Introduction

Parkinson’s disease (PD) is the most common chronic and progressive neurodegenerative movement disorder. Although it primarily affects the motor nervous system, sensory symptoms may also be present [1]. According to the World Health Organization, PD affects 1–2% of individuals over the age of 65 years, with prevalence rising to 2–3% among those over 85 years [1–6].

Visual symptoms can emerge as early manifestations of PD, affecting both dorsal and ventral visual pathways. These changes may result in visual processing deficits, including impaired spatial perception, stereoacuity, colour discrimination, visual attention and facial recognition. Notably, contrast sensitivity is often significantly affected [7–11].

Global contrast perception, typically evaluated using the contrast sensitivity function (CSF), provides a valuable measure of visual quality by assessing both optical and neural components of the visual system [12]. CSF is an effective indicator of visual impairment, allowing clinicians to detect diseases which are most prevalent in individuals over 50 years of age [13]. Visual performance is frequently diminished in individuals with abnormal vision conditions, especially under either mesopic or glare conditions, as compared to photopic levels [14]. Even healthy older adults commonly report visual difficulties under low illumination [15, 16]. CSF can also be an effective tool for tracking disease progression and monitoring treatment efficacy.

Multiple studies have demonstrated that individuals with PD experience a marked reduction in photopic contrast sensitivity, often involving intermediate and high spatial frequencies [17–20]. This deficit may be attributed to dysfunctions in the magnocellular pathway, which processes spatial-temporal visual stimuli. The degeneration of dopaminergic neurons in the retina, as well as disruptions in the basal ganglia and substantia nigra, contribute to impaired contrast perception [21]. Moreover, the progression of PD and associated cognitive decline have been identified as key predictors of visual impairments, including contrast sensitivity deficits [22]. Understanding these visual changes is crucial, as they may serve as early indicators of disease severity and influence the ability of the person to perform activities of daily living, such as driving [23].

The aim of the present prospective and comparative study was to assess, for the *first time*, the CSF in participants with PD under six different illumination conditions, while considering the stage of the disease. This approach is particularly relevant because visual perception in real-world environments requires the integration of information across a wide range of spatial frequencies under variable lighting conditions, including low luminance and glare. Therefore, assessing CSF under different illuminations may reveal visual deficits that are not evident under standard photopic clinical testing, and may help to identify illumination conditions that are especially sensitive to PD-related visual dysfunction. Such conditions could provide an optimal clinical test setting for monitoring disease progression and may contribute to the development of functional visual biomarkers [24]. Thus, understanding how different lighting environments impact visual performance in PD can offer deeper insight into the underlying visual deficits associated with the disease, support the development of improved clinical diagnostic and follow-up tools and ultimately inform strategies aimed at enhancing patient care and quality of life [24].

## Material and Methods

### Participants

Age-matched healthy controls (control group, CG) and individuals with PD (PD group, PG) were recruited for the study. Inclusion criteria included the absence of ocular or systemic diseases affecting vision, no neurodegenerative diseases other than PD, no medications affecting vision, the ability to follow the experimental tests and written informed consent to participate. Control participants were matched by age and sex. PD participants were required to have a neurologist-confirmed diagnosis, classification according to the Hoehn and Yahr scale [25], no prior vision therapy and no diagnosis of dementia. Participants in Hoehn and Yahr stages IV and V were excluded, as one of the inclusion criteria was the ability to understand and perform the tests, which may be compromised at these advanced stages due to the frequent presence of cognitive impairment. Consequently, no formal cognitive tests were administered. Participants in Hoehn and Yahr stage I were treated with levodopa, including some who were newly diagnosed, whereas participants in stages II and III were treated with levodopa and/or dopaminergic agonists and monoamine oxidase B inhibitors, specifically rasagiline. No participants being treated with deep-brain stimulation or infusion pump therapies were included. No untreated participants were included in the study. Only participants who underwent a comprehensive neurological and ophthalmological examination were included in the study.

An estimated sample size of approximately 80 participants per group was calculated using a standard sample size estimation formula, assuming a 95% confidence level and a margin of error of 0.05. A non-probability sampling strategy was applied, and a larger sample was ultimately recruited to allow for potential exclusions during age-matching. Controls were recruited via university channels and local associations, while PD participants were recruited through the Association Parkinson Valencia and Arnau Vilanova-Llíria Hospital of Valencia, Spain. All participants provided written informed consent following the ethical guidelines of the Declaration of Helsinki. The study was approved by an independent ethical review board at each institution, and was registered at ClinicalTrials.gov (Identifier: NCT06032130, registered on September 29, 2023).

### Procedure

This observational, analytical, *prospective*, cross-sectional study involved a detailed medical history evaluation for each participant to ensure they met the inclusion criteria. Participants underwent trial frame refraction, best-corrected visual acuity (BCVA) and CSF under six different illumination conditions. First, visual acuity was measured with the habitual optical correction. If the visual acuity subsequently improved when viewing through a pinhole, the refraction was updated with the Maximum Plus to Maximum Visual Acuity criterion [26]. If there was no further improvement, then the refraction of the participant was not updated.

CG participants were assessed in a single session at the University of Valencia, while PD participants were assessed at their home institution. Tests were conducted in quiet, well-lit, windowless rooms, with each session lasting about 45 min. CSF was first measured under mesopic and then under photopic conditions. All procedures were performed by the same investigator to ensure consistency and were measured once. Rest periods were provided as needed.

### Hoehn and Yahr Scale

Each patient in the PG had their Hoehn and Yahr status [25] provided by the institution of choice. The Hoehn and Yahr scale classifies PD progression into five stages:

1. Stage I: Recent diagnosis of unilateral involvement and minimal functional impairment.
2. Stage II: Bilateral or midline involvement without vestibular dysfunction.
3. Stage III: Moderate bilateral involvement with impaired balance and postural reflexes, leading to mild-to-moderate disability.
4. Stage IV: Increased dependence, although the individual can still walk or stand unaided.
5. Stage V: Severe disability with high dependence, often confined to bed or wheelchair without aid.

### Apparatus

BCVA was assessed using an Early Treatment of Diabetic Retinopathy Study visual acuity chart (Precision Vision, precision-vision.com) [27] at 4 m with an illuminance of 106 lx in a lighted room (650 lx). The logMAR scale was used, and no pupil dilation was performed. CSF measurements were performed at 4 m using the Functional Acuity Contrast Test (Stereo Optical Co., Inc., stereooptical.com) [28], integrated within the Optec 6500 Functional Vision Analyzer device (stereooptical.com). It was employed under different illumination conditions (Table 1). Luminance levels were measured using a Spectra Scan PR655 tele-spectrophotometer (photoresearch.com) at 1° intervals. Light-emitting diodes around the target were used to create glare.

To measure the CSF, participants were instructed to report the inclination or tilt of each stimulus, looking at each row from left to right and from top to bottom and having the choice of left leaning, right leaning, vertical or non-detectable. The last response seen by the participant was recorded [29].

**Table 1:** Illumination and glare testing conditions with corresponding LED luminance.

Illumination condition	Glare condition	LED luminance (cd/m^2^)	Glare setup
Photopic (85 cd/m²)	None	N/A	No glare
Photopic (85 cd/m²)	G1	642.50	12 LEDs: 3 left, 3 right, 3 top, 3 bottom
Photopic (85 cd/m²)	G2	12.51	Same as G1
Mesopic (3 cd/m²)	None	N/A	No glare
Mesopic (3 cd/m²)	G1	90.81	Same as G1
Mesopic (3 cd/m²)	G2	2.30	Same as G1

*LED* light-emitting diode.

G1 for both photopic and mesopic conditions was categorised as discomfort glare, whereas G2 for both conditions was classified as disability glare [30]. Spatial frequencies of 1.5, 3, 6, 12 and 18 cycles per degree (cpd) were assessed using the ascending method of limits. A 10-min light adaptation period was employed before conducting the measurements.

Illumination levels were monitored using a Konica Minolta Chroma Meter CL-200 photometer (sensing.konicaminolta.eu), equipped with an integrating-sphere sensor. Calibration of the instruments was verified prior to measurements. Participants underwent testing while seated on a comfortable chair at a table in a darkened room (5 lx measured at the centre of the room) to minimise stray reflections. The Functional Acuity Contrast Test was conducted monocularly, with the sensorially dominant eye of each participant determined using the Red Filter Test [31]. The non-dominant eye was fully occluded.

### Statistical Analysis

Data were analysed using IBM SPSS software (version 31.0; ibm.com/products/spss-statistics). Variables analysed included age, sex, disease stage and CSF in six different illuminations, i.e., photopic, photopic G1, photopic G2, mesopic, mesopic G1 and mesopic G2. The index of contrast sensitivity (ICS) was calculated according to Haughom and Strand [32]. The ICS is an index for the evaluation of overall CSF and comparison of different participant groups [33]. The ICS is a simple linear weighting function considering that the sensitivity of the eye peaks at 6 cpd:

1 $${{{\rm{ICS}}}}={{{\rm{dCSF}}}}(1.5)+2\cdot {{{\rm{dCSF}}}}(3)+3\cdot {{{\rm{dCSF}}}}(6)+2\cdot {{{\rm{dCSF}}}}(12)+{{{\rm{dCSF}}}}(18)$$

where dCSF(*f*) refers to the deviation of the measured CSF value and the median value of spatial frequency *f*:

2 $${{{\mathrm{dCSF}}}}_{i}(f)\,=\,{{{\mathrm{CSF}}}}_{i}(f)\, - \,{{{\mathrm{Median}}}}\,({{{\mathrm{CSF}}}}(f))$$

which reduces age-related bias. The rationale behind the ICS is to reduce global differences between CSF measurements obtained under different conditions [32].

Descriptive statistics were used to summarise central tendency and dispersion. Assumptions of normality were assessed to inform the choice of parametric analyses. Contrast sensitivity data were analysed using a mixed repeated-measures analysis of variance (ANOVA), with group (PG, CG) as a between-subjects factor and illumination (photopic, photopic glare 1, photopic glare 2, mesopic, mesopic glare 1 and mesopic glare 2) and spatial frequency (1.5, 3, 6, 12 and 18 cpd) as within-subject factors. To examine the effect of disease severity, an additional mixed repeated-measures ANOVA was conducted within the PG, with disease stage (Hoehn and Yahr stages I, I and III) as a between-subjects factor. Given the known association between age and disease stage, analyses examining disease stage were repeated, including age as a covariate (ANCOVA), to account for potential age-related confounding effects.

The ICS was analysed using separate mixed repeated-measures ANOVAs, with illumination as a within-subject factor and either group (PG vs CG) or disease stage (Hoehn and Yahr I-III) as between-subjects factors. Additionally, age was included as a covariate in disease-stage analyses of ICS. Where significant interactions were observed, follow-up between-group comparisons were conducted using independent-samples *t*-tests. To control for inflation of type I errors due to multiple comparisons, *p* values were adjusted using the Holm–Bonferroni procedure. Effect sizes for ANOVA effects were expressed as partial eta squared (*ηp*²), while effect sizes for pairwise comparisons were quantified using Cohen’s *d*. Statistical significance was set at *α* = 0.05.

## Results

Healthy controls (CG: *n* = 96, 47 males (49%), mean 62 ± 7 years, BCVA: −0.02 ± 0.06 logMAR) and participants with PD (PG: *n* = 76, 43 males (57%), mean 68 ± 8 years, BCVA: 0.02 ± 0.14 logMAR) were recruited for the study. After age-matching, the final sample included the same number of healthy controls (CG: *n* = 61, 32 females [52%], mean age 66 ± 6 years, BCVA: −0.02 ± 0.06 logMAR) and participants with PD (PG: *n* = 61, 27 females [44%], mean age 66 ± 6 years, BCVA: 0.02 ± 0.14 logMAR). No statistically significant differences were found between groups in terms of age (*p* = 0.90) or sex distribution (*p* = 0.40). In contrast, a small but statistically significant difference was observed in BCVA between groups (*p* = 0.001).

### Comparison of CS at Each Spatial Frequency Between PD Participants and Controls

A mixed repeated-measures ANOVA was conducted with group (PG, CG) as a between-subjects factor and illumination (six levels) and spatial frequency (five levels) as within-subject factors. Analysis revealed a significant main effect of illumination, *F*(5,120) = 303.5, *p* < 0.001, *ηp*² = 0.7, indicating that contrast sensitivity varied across illumination conditions. A significant main effect of spatial frequency was also observed, *F*(4,120) = 473.3, *p* < 0.001, *ηp*² = 0.8.

A significant between-subjects effect of group was found, *F*(1,120) = 27.5, *p* < 0.001, *ηp*² = 0.2, indicating overall differences in contrast sensitivity between PD and controls when averaged across experimental conditions. Significant interactions were observed between group and illumination, *F*(5,120) = 10.5, *p* < 0.001, *ηp*² = 0.08, between group and spatial frequency, *F*(4,120) = 19.0, *p* < 0.001, *ηp*² = 0.1 and between illumination and spatial frequency, *F*(20,120) = 68.0, *p* < 0.001, *ηp*² = 0.4. Importantly, a significant three-way interaction between group, illumination and spatial frequency was also found, *F*(20,120) = 5.19, *p* < 0.001, *ηp*² = 0.04. This three-way interaction indicates that group differences in contrast sensitivity depended jointly on the illumination condition and spatial frequency.

To characterise the significant three-way interaction, post-hoc between-group comparisons were conducted for each combination of illumination condition and spatial frequency. Independent-samples *t*-tests were performed, with p values adjusted for multiple comparisons using the Holm–Bonferroni procedure. Effect sizes were quantified using Cohen’s d; *p* values < 0.001 were conservatively treated as =0.001 for the purpose of adjustment.

These analyses revealed that participants with PD exhibited significantly reduced contrast sensitivity compared with controls under specific combinations of illumination and spatial frequency, with effect sizes ranging from moderate to large. After adjustment for multiple comparisons, significant between-group differences were observed, primarily at mid-to-high spatial frequencies under photopic and glare conditions and at mid spatial frequencies under mesopic illumination. No significant group differences were found at the lowest spatial frequency (1.5 cpd) under photopic illumination or at the highest spatial frequency (18 cpd) under mesopic illumination. Detailed results of the between-group post-hoc comparisons for all illumination conditions and spatial frequencies are provided in Table 2.

**Table 2:** Between-group comparisons of contrast sensitivity across illumination conditions and spatial frequencies.

Illumination	SF (cpd)	PD (mean ± SD)	Controls (mean ± SD)	*t*(df)	*p*	Cohen’s *d*
Photopic	1.5	32.57 ± 16.10	34.52 ± 12.87	−0.74 (120)	0.50	−0.13
	3	51.34 ± 25.24	67.74 ± 25.90	−3.54 (120)	**<0.001**	−0.64
	6	44.66 ± 25.45	74.10 ± 38.79	−4.96 (120)	**<0.001**	−0.90
	12	17.74 ± 16.81	30.16 ± 15.38	−4.26 (120)	**<0.001**	−0.77
	18	4.39 ± 6.37	7.67 ± 5.55	−3.03 (120)	0.003	−0.55
Photopic glare 1	1.5	38.05 ± 19.65	45.26 ± 18.06	−2.11 (120)	0.04	−0.38
	3	57.74 ± 30.21	77.48 ± 29.70	−3.64 (120)	**<0.001**	−0.66
	6	47.82 ± 29.85	78.57 ± 34.65	−5.25 (120)	**<0.001**	−0.95
	12	17.08 ± 15.79	28.92 ± 16.72	−4.02 (120)	**<0.001**	−0.73
	18	4.70 ± 5.27	8.52 ± 5.47	−3.93 (120)	**<0.001**	−0.71
Photopic glare 2	1.5	27.97 ± 13.52	37.30 ± 11.49	−4.11 (120)	**<0.001**	−0.74
	3	44.31 ± 21.79	67.05 ± 25.39	−5.31 (120)	**<0.001**	−0.96
	6	36.38 ± 26.36	61.49 ± 28.33	−5.07 (120)	**<0.001**	−0.92
	12	12.10 ± 11.73	23.18 ± 14.76	−4.59 (120)	**<0.001**	−0.83
	18	3.74 ± 7.71	6.69 ± 5.18	−2.48 (120)	0.01	−0.45
Mesopic	1.5	30.48 ± 18.95	36.49 ± 14.37	−1.98 (120)	0.05	−0.36
	3	39.46 ± 23.24	47.36 ± 19.30	−2.04 (120)	0.04	−0.37
	6	20.79 ± 21.58	32.36 ± 15.96	−3.37 (120)	**0.001**	−0.61
	12	5.36 ± 8.64	6.98 ± 6.74	−1.16 (120)	0.30	−0.21
	18	0.87 ± 2.62	1.05 ± 2.15	−0.42 (120)	0.70	−0.08
Mesopic glare 1	1.5	25.69 ± 16.34	35.15 ± 12.57	−3.59 (120)	**<0.001**	−0.65
	3	31.69 ± 18.71	48.89 ± 22.10	−4.64 (120)	**<0.001**	−0.84
	6	16.36 ± 14.78	30.75 ± 16.83	−5.02 (120)	**<0.001**	−0.91
	12	3.72 ± 6.70	7.08 ± 8.09	−2.50 (120)	0.01	−0.45
	18	0.51 ± 2.39	0.85 ± 1.91	−0.88 (120)	0.40	−0.16
Mesopic glare 2	1.5	11.31 ± 10.12	18.05 ± 10.81	−3.55 (120)	**<0.001**	−0.64
	3	16.16 ± 13.47	25.80 ± 11.61	−4.23 (120)	**<0.001**	−0.77
	6	7.77 ± 11.84	16.38 ± 14.55	−3.58 (120)	**<0.001**	−0.65
	12	1.18 ± 3.74	2.34 ± 4.32	−1.59 (120)	0.10	−0.29
	18	0.00 ± 0.00	0.23 ± 1.04	−1.73 (120)	0.09	−0.31

Values are mean ± standard deviation. *p* values are two-tailed. Effect size is reported as Cohen’s *d*. Significant comparisons surviving Holm–Bonferroni correction are indicated in bold.

*cpd* cycles per degree, *PD* Parkinson’s Disease, *SF* spatial frequency, *SD* standard deviation.

**Fig. 1:**
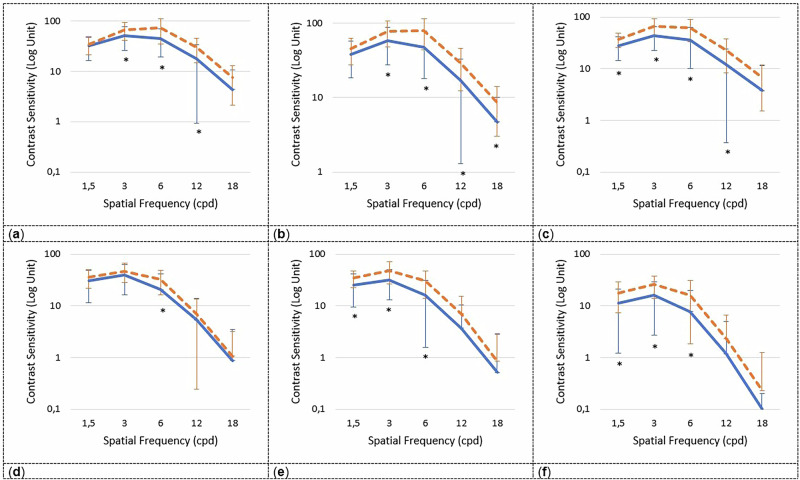
Contrast sensitivity function (CSF) comparison (mean ± standard deviation) between the Parkinson’s Disease group (PG) (blue solid line) and the control group (CG) (orange dashed line) under different illumination conditions: **a** photopic; **b** photopic with glare level 1 (G1); **c** photopic with glare level 2 (G2); **d** mesopic; **e** mesopic with G1; and **f** mesopic with G2. Asterisks indicate statistically significant differences between groups (*p* < 0.05). cpd cycles per degree.

Figure 1 presents a comparison between the PG and CG across all illumination conditions. The findings show that at every illumination level, the PG exhibited significantly lower CSF than the CG. These descriptive differences are consistent with the significant group × illumination × spatial frequency interaction identified in the mixed-model analysis. Within both groups, the ranking of illumination conditions from highest to lowest CSF was as follows: photopic G1, photopic, photopic G2, mesopic, mesopic G1 and mesopic G2.

For ICS, a mixed repeated-measures ANOVA was conducted with illumination as a within-subject factor and group as a between-subject factor. A significant main effect of illumination was observed, *F*(5,120) = 2.8, *p* = 0.04, *ηp*² = 0.02. No significant main effect of group was found, *F*(1,120) = 0.02, *p* = 0.90, *ηp*² < 0.001. However, a significant interaction between group and illumination was detected, *F*(5,120) = 9.8, *p* < 0.001, *ηp*² = 0.08. Follow-up between-group comparisons indicated that group differences in ICS emerged under specific illumination conditions, despite the absence of an overall group effect.

Post-hoc between-group comparisons of ICS were performed for each illumination condition, with *p* values adjusted using the Holm–Bonferroni procedure. After correction, a significant group difference was observed only under mesopic illumination, with participants with PD showing higher ICS values than controls (*t*(120) = 3.59, *p*_adj_ = 0.006, Cohen’s *d* = 0.7). No significant between-group differences were detected under photopic or glare conditions (all *p*_adj_ ≥ 0.40). Detailed results are provided in Table 3.

**Table 3:** Between-group comparisons of index of contrast sensitivity (ICS) across illumination conditions.

Illumination	PG (mean ± SD)	CG (mean ± SD)	*t*(df)	*p*	*p*_adj_	Cohen’s *d*
Photopic	4.10 ± 152.16	50.30 ± 187.27	−1.50 (120)	0.10	0.60	−0.27
Photopic glare 1	16.85 ± 181.13	62.30 ± 193.10	−1.34 (120)	0.20	0.70	−0.24
Photopic glare 2	26.66 ± 143.25	16.92 ± 152.46	0.36 (120)	0.70	<0.99	0.07
Mesopic	**49.34** ± **127.02**	**−22.69** ± **91.93**	**3.59 (120)**	**<0.001**	**0.006**	**0.65**
Mesopic glare 1	12.10 ± 89.34	8.20 ± 101.46	0.23 (120)	0.80	>0.99	0.04
Mesopic glare 2	20.31 ± 69.25	−3.30 ± 73.77	1.82 (120)	0.07	0.40	0.33

Values are mean ± standard deviation. *p* values are two-tailed. Effect size is reported as Cohen’s *d*. Significant comparisons surviving Holm–Bonferroni correction are indicated in bold.

*CG* control group, *p*_*adj*_ *p*-values adjusted using the Holm–Bonferroni procedure, *PG* group with Parkinson’s disease, *SD* standard deviation.

Taken together, the present findings indicate that group differences in visual performance are highly dependent on illumination and spatial frequency. For contrast sensitivity, the significant three-way interaction between group, illumination and spatial frequency revealed that participants with PD exhibited selectively reduced sensitivity at mid-to-high spatial frequencies, under photopic and glare conditions. In contrast, ICS did not show a general group effect but rather demonstrated a condition-specific difference, with lower values in the PG observed exclusively under mesopic illumination after correction for multiple comparisons. These results highlight the importance of considering both illumination and spatial frequency when characterising visual function in PD, and underscore the context-dependent nature of group differences.

### Comparison of CS at Each Spatial Frequency Between PD Disease Stage

**Fig. 2:**
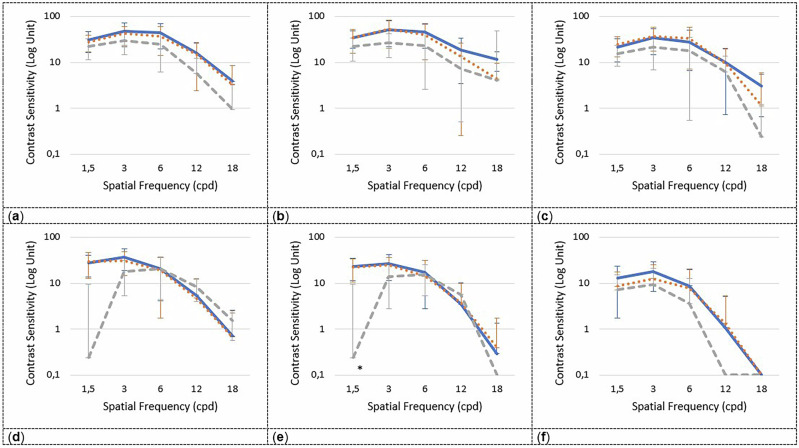
Contrast sensitivity function (CSF) comparison (mean ± standard deviation) between Parkinson’s disease (PD) stage groups according to the Hoehn and Yahr classification (Stage I: blue solid line; Stage II: orange dotted line; Stage III: grey dashed line) under different illumination conditions: **a** photopic; **b** photopic with glare level one (G1); **c** photopic with glare level two (G2); **d** mesopic; **e** mesopic with G1 and **f** mesopic with G2. cpd cycles per degree.

The PG was stratified into three stages: Stage I (*n* = 14), with 8 males (57%), mean age 66 ± 11 years and a BCVA of −0.02 ± 0.08 logMAR; Stage II (*n* = 45), with 28 males (62%), mean age 67 ± 8 years and BCVA of 0.00 ± 0.14 logMAR and Stage III (*n* = 17), with 7 males (41%), mean age 71 ± 6 years and BCVA of 0.04 ± 0.18 logMAR. Figure 2 presents a comparison of CSF across Hoehn and Yahr stages I, II and III in the PG for all illumination conditions. Visual inspection suggests a progressive reduction in CSF with increasing disease stage across illumination conditions; however, these patterns were formally evaluated using mixed-model analyses as reported below.

In the unadjusted mixed repeated-measures ANOVA, a significant main effect of illumination was observed, *F*(5,73) = 88.4, *p* < 0.001, *ηp*² = 0.6, indicating that contrast sensitivity differed across illumination conditions. A significant main effect of spatial frequency was also found, *F*(4,73) = 133.8, *p* < 0.001, *ηp*² = 0.7. A significant between-subjects main effect of disease stage was present, *F*(2,73) = 4.6, *p* = 0.01, *ηp*² = 0.1, indicating overall differences in contrast sensitivity across disease stages.

With respect to interaction effects, the illumination × disease stage interaction reached significance, *F*(10,73) = 2.0, *p* = 0.04, *ηp*² = 0.05, suggesting that stage-related differences varied across illumination conditions. The spatial frequency × disease stage interaction was also significant, *F*(8,73) = 3.8, *p* < 0.001, *ηp*² = 0.09. A robust illumination × spatial frequency interaction was observed, *F*(20,73) = 18.6, *p* < 0.001, *ηp*² = 0.2, indicating that the effect of spatial frequency depended on illumination. Finally, the three-way interaction between illumination, spatial frequency and disease stage was significant, *F*(40,73) = 1.8, *p* = 0.003, *ηp*² = 0.05.

When age was included as a covariate, the main effects of illumination and spatial frequency remained significant, *F*(5,72) = 8.0, *p* < 0.001, *ηp*² = 0.1 and *F*(4,72) = 23.1, *p* < 0.001, *ηp*² = 0.2, respectively. The main effect of disease stage was no longer statistically significant after adjustment for age, *F*(2,72) = 3.1, *p* = 0.05, *ηp*² = 0.08. Age showed a significant main effect, *F*(1,72) = 20.5, *p* < 0.001, *ηp*² = 0.2, thus confirming a substantial influence of age on contrast sensitivity.

No significant illumination × disease stage interaction was observed, *F*(10,72) = 1.5, *p* = 0.10, *ηp*² = 0.04. However, significant interactions were found between illumination and age, *F*(5,72) = 2.7, *p* = 0.02, *ηp*² = 0.04 and between spatial frequency and age, *F*(4,72) = 11.2, *p* < 0.001, *ηp*² = 0.1. The interaction between spatial frequency and disease stage remained significant after age adjustment, *F*(8,72) = 2.6, *p* = 0.01, *ηp*² = 0.07, as did the illumination × spatial frequency interaction, *F*(20,72) = 1.9, *p* = 0.009, *ηp*² = 0.03. The three-way interaction between illumination, spatial frequency and disease stage also remained significant, *F*(40,72) = 1.6, *p* = 0.01, *ηp*² = 0.04. In contrast, the illumination × spatial frequency × age interaction was not significant, *F*(20,72) = 0.9, *p* = 0.60, *ηp*² = 0.01.

For ICS, in the unadjusted mixed repeated-measures ANOVA, a significant main effect of illumination was observed, *F*(5,73) = 4.4, *p* < 0.001, *ηp*² = 0.06, indicating that ICS varied with illumination. A significant between-subjects main effect of disease stage was also found, *F*(2,73) = 4.5, *p* = 0.02, *ηp*² = 0.1. In addition, the illumination × disease stage interaction was significant, *F*(10,72) = 2.3, *p* = 0.01, *ηp*² = 0.06, suggesting that stage-related differences in ICS depended on illumination condition.

After inclusion of age as a covariate, the main effect of illumination remained significant, *F*(5,72) = 3.0, *p* = 0.01, *ηp*² = 0.04. In contrast, the main effect of disease stage was no longer statistically significant, *F*(2,72) = 2.9, *p* = 0.06, *ηp*² = 0.08. Age showed a significant main effect on ICS, *F*(1,72) = 20.3, *p* < 0.001, *ηp*² = 0.2. Accordingly, the interpretation of disease stage effects on ICS was based on the age-adjusted model. The interaction between illumination and age was significant, *F*(5,72) = 3.3, *p* = 0.006, *ηp*² = 0.04, indicating that the effect of illumination on ICS varied as a function of age. The illumination × disease stage interaction was no longer significant after adjustment for age, *F*(10,72) = 1.76, *p* = 0.07, *ηp*² = 0.05.

## Discussion

To the best of the available and current knowledge of the authors, this is the *first study* to assess CSF and provide ICS data under six different illumination conditions in participants with PD, while considering disease stage and comparing with an age-matched CG.

The present study revealed two main conclusions. The *first* was that participants with PD demonstrated an overall reduction of CSF across most illumination conditions as compared with the controls, with particularly pronounced deficits under mesopic conditions. The *second* was that this reduction in CSF showed a trend to vary with advancing disease stage, although this effect was attenuated after controlling for age, and the ICS did not correlate significantly with disease progression.

The absence of a significant effect of disease stage at the highest spatial frequency assessed (18 cpd) reflects a floor effect. Contrast sensitivity at high spatial frequencies was already markedly reduced in participants at early disease stages, leaving little range for further deterioration to be detected as the disease progressed. From a clinical perspective, this finding suggests that high spatial frequencies may have limited utility for monitoring disease progression in PD, as impairments appear early and reach a performance floor. In contrast, mid spatial frequencies, which retain greater sensitivity to stage-related changes, may represent more informative targets for clinical assessment and longitudinal monitoring.

The CSF values for the PD participants obtained in the present study are comparable to those reported in previous investigations. A marked reduction in *photopic* CSF has been reported among PD participants, particularly affecting mid and high spatial frequencies [17–20]. Importantly, the findings of the present study extend this evidence by demonstrating that CSF impairment in PD is not confined to photopic conditions, but is also evident under mesopic and glare conditions. This aligns with a prior investigation reporting an overall CSF decline under *photopic* conditions [34], which was attributed to the role of dopaminergic retinal ganglion cells in regulating the diurnal variation in CSF [35]. These cells are compromised in PD, as evidenced by the improvement in CSF following the administration of dopaminergic medication [36].

The results of the current study are in agreement with previous research, but further extend these findings by demonstrating that CSF impairment in PD is not limited to photopic conditions, but also occurs under adverse visual environments, such as mesopic and glare conditions. This widespread reduction in CSF supports the hypothesis suggesting involvement of both magnocellular and parvocellular visual pathways in PD participants [21].

The visual impairments observed in the present study provide a plausible explanation for the range of functional visual difficulties commonly reported by individuals with PD. Impaired performance under low illumination, such as negotiating stairs or curbs, as well as reduced ability to drive at night and participate in leisure activities under dim lighting conditions, corresponds with the documented decline in contrast sensitivity across photopic, mesopic and glare environments. The particularly pronounced deficit observed under mesopic conditions may reflect the increased reliance on dopaminergic retinal mechanisms and rod-cone interactions in intermediate luminance levels, which are known to be vulnerable in PD. Additionally, difficulties related to glare from vehicle headlights and street lighting, challenges in reading against coloured or grey backgrounds, the need to increase the brightness of electronic devices and compromised adaptation to rapid changes in ambient light, e.g., when entering or exiting tunnels or rooms, further illustrate the extensive impact of contrast sensitivity deficits on everyday visual tasks [37]. Collectively, these impairments contribute to a reduction in the quality of life, emphasising the necessity to incorporate detailed and specialised visual function assessment and management in the comprehensive care of persons with PD [38].

From a clinical perspective, the present findings indicate that contrast sensitivity assessment in PD should extend beyond standard photopic conditions. Mesopic conditions revealed the largest and most consistent deficits, suggesting that these settings may be the most sensitive for detecting visual dysfunction and monitoring disease progression. In particular, assessment at mid spatial frequencies (around 6 cpd), where contrast sensitivity normally peaks, appears especially informative. Accordingly, contrast sensitivity testing under mesopic and glare conditions may provide greater clinical value than photopic testing alone when contrast sensitivity is considered as a clinically relevant tool for characterising visual dysfunction in PD, and for exploring its use as a functional biomarker of disease severity and progression.

It should be noted that direct comparison of contrast sensitivity is feasible using the CSF, as its assessment requires 30 individual measurements per eye, covering five spatial frequencies under six distinct illumination conditions (mesopic, mesopic glare 1, mesopic glare 2, photopic, photopic glare 1 and photopic glare 2). However, this multidimensional complex dataset poses considerable challenges for statistical analysis and intersubject comparisons.

To address this complexity, Haughom and Strand [32] proposed the ICS, which condenses CSF data into a single numerical value per illumination condition. The ICS accounts for the perceptual weighting of spatial frequencies, with particular emphasis on 6 cpd, where visual sensitivity typically peaks. As such, the ICS provides a more concise and functionally relevant summary measure, thus facilitating comparison across subjects and conditions. Consistent with this rationale, the present findings indicate that ICS may be less sensitive to subtle, stage-related changes in visual function once age-related effects are taken into account.

Nonetheless, this leads to the limitations of the present study. Firstly, although the overall sample size was adequate for comparisons between participants with PD and healthy controls, the subgroup sample sizes for disease stage analyses were more limited. Future research would benefit from the inclusion of a larger and more diverse cohort, particularly with an increased representation of participants in Hoehn and Yahr stages I and III, to improve generalisability. Accordingly, findings related to disease stage should be interpreted with caution, and further studies with larger, stage-specific samples are warranted. In addition, given the repeated-measures design, a potential limitation is the presence of order effects, as participants were assessed sequentially under multiple illumination conditions, which may have introduced familiarity or learning effects across testing sessions. Future studies should consider randomising or counterbalancing the order of illumination conditions to mitigate this possibility. Moreover, longitudinal studies are warranted to evaluate changes in contrast sensitivity over time and to elucidate the trajectory of CSF deterioration as PD progresses. Such studies may also help identify early markers of visual dysfunction, enabling earlier and more targeted interventions.

One notable limitation of the present study is the exclusive use of the Hoehn and Yahr scale to classify disease severity. While this staging system is widely recognised and clinically accepted [29], its dependence on motor symptoms alone may not fully capture the complex, multidimensional nature of PD. Incorporating additional assessments, such as the Unified Parkinson’s Disease Rating Scale [39], the Mini-Mental State Examination [40] and the Montreal Cognitive Assessment [41] could provide a more comprehensive evaluation of the interrelationships between motor, cognitive and visual impairments in PD. In addition, although BCVA differed significantly between groups, this difference was considered to be disease-related, as BCVA is closely linked to high spatial frequency contrast sensitivity under photopic conditions, which is known to be affected in PD [42]. Such multidimensional assessment may also help disentangle disease-related visual changes from age-associated effects. Future research should consider the use of these complementary measures, as well as other scales, to explain further the multifaceted progression of the disease.

In conclusion, to the best of our current knowledge, this is the first study to assess CSF systematically and report related ICS data under six controlled illumination conditions in participants with PD, across different stages of disease progression and in comparison with age-matched healthy controls. These findings may contribute to a more comprehensive understanding of visual function across different stages of the disease. Importantly, the present results suggest that while contrast sensitivity deficits are robust in PD, stage-related differences are partially influenced by age. The new results also provide novel insights into the stage-dependent visual processing deficits in PD. Lastly, such novel insights may have significant implications for both developing clinical assessment protocols and establishing vision-based biomarkers for disease monitoring.

## Data Availability

The data that support the findings of this study are not publicly available due to privacy and ethical restrictions relating to the participants involved, but are available from the corresponding author upon reasonable request.
